# Health Behaviors and Health Literacy: Questing the Role of Weak Social Ties Among Older Persons in Rural and Urban Ghana

**DOI:** 10.3389/fpubh.2022.777217

**Published:** 2022-02-28

**Authors:** Padmore Adusei Amoah, John Musalia, Kwaku Abrefa Busia

**Affiliations:** ^1^Department of Applied Psychology, School of Graduate Studies, Institute of Policy Studies, Lingnan University, Tuen Mun, Hong Kong SAR, China; ^2^Department of Sociology and Criminology, Western Kentucky University, Bowling Green, KY, United States; ^3^Department of Sociology and Social Policy, Lingnan University, Tuen Mun, Hong Kong SAR, China

**Keywords:** health behaviors, health literacy, social capital, weak ties, older persons, rural, urban, Ghana

## Abstract

**Background:**

Older persons are one of the most vulnerable groups as regards low health literacy. However, little is known about the extent of limitations and multi-faceted nature of their health literacy, such as its characteristics and social and geographical dimensions. Additionally, most existing studies have predominantly treated health literacy as a risk factor of health and wellbeing of older persons as opposed to an outcome that must be pursued.

**Objectives:**

This study investigated the moderating role of weak social ties (bridging social capital) in the relationship between health behaviors, such as smoking, alcohol intake, voluntary body check-up and physical exercise, and health literacy among older persons in rural and urban Ghana.

**Methods:**

Data was drawn from a cross-sectional survey comprising 522 respondents across five administrative regions in Ghana. Ordinary Least Squares regression technique was used to analyse the data.

**Results:**

Older persons in urban areas had higher health literacy [Mean/Standard deviation (SD) = 9.1/4.1 vs. 10.1/4.2] as well as higher bridging social capital (Mean/SD = 2.0/1.2 vs. 1.6/0.9) than their rural counterparts. Bridging social capital was negatively associated with the health literacy of urban residents (*B* = −0.997, *p* < 0.01). We found evidence that smoking (*B* = −0.787, *p* < 0.05) and undertaking physical activities (*B* = 0.812, *p* < 0.01) were associated with health literacy of older persons in rural areas. Having voluntary body check-ups (*B* = 0.155, *p* < 0.01) was associated with health literacy in urban areas. Bridging social capital negatively moderated the association of smoking with health literacy in rural areas (*B* = −5.032, *p* < 0.01), but it instead positively modified the relationship between alcohol intake and health literacy in urban areas (*B* = 0.185, *p* < 0.05).

**Conclusion:**

For policymakers and practitioners aiming to promote older persons' health literacy as a public health asset at individual and community levels, an important starting point to achieving such goals is to understand the fundamental indicators (e.g., health behaviors) and the role that social and geographical factors play in shaping their health literacy.

## Introduction

Health literacy (HL) “entails people's knowledge, motivation and competence to access, understand, appraise, and apply health information in order to make judgments and take decisions in everyday life concerning healthcare, disease prevention and health promotion to maintain or improve quality of life during the life course” [(1), p. 3]. It is predominantly considered a primary determinant of health (1–3). Low HL is associated with poor health service utilization practices (4, 5), increased likelihood of deleterious health behaviors (6–8), poor health status and chronic illnesses (9–11), higher health care costs due to higher hospitalization rates (5, 10, 12), and increased likelihood of mortality (13–15).

Older persons are considered one of the most at-risk populations regarding low health literacy (6, 12). Low health literacy among older persons is widely attributed to inevitable aging-related cognitive decline and high rates of illiteracy (5, 6, 15–17). However, research on the extent of limitations and multi-faceted nature of older persons' health literacy in terms of its characteristics, confounders, and social and geographical dimensions are underdeveloped (3, 18–21). Extant evidence from Ghana and other countries indicate that rural and urban residents have different levels of health literacy, and their social environments (e.g., social capital) are critical to their health-related well-being (22–24). This is because the physical and social environment that people find themselves can influence how they seek to translate and apply health information (25–28). Such influences on health-related knowledge and its application often lead to an inconsistent conceptualization of health literacy (29). Unfortunately, the dynamics of how factors such as different forms of social capital affect health literacy, its potential determinants and the geographical dimensions to such influence are less understood among older persons.

Moreover, health literacy research evidence among older persons and theoretical positions on health literacy are dominated by positions that present health literacy as a risk or causal factor in health and welbeing (2, 30–32). Health literacy research and practice that leans toward the risk factor perspective focuses on the relationship between low literacy, health and interventions to mitigate the effects of low literacy, which position health literacy as a potential risk factor for poor health outcomes that need to be managed as part of health and social care provisions (30). This approach is usually criticized for ignoring the need to understand health literacy as an asset that health systems and gerontological researchers must pursue. Consideration of health literacy as an asset— “an outcome to health education and communication that supports greater empowerment in health decision-making” [(30), p. 2,074], offers a needed impetus to design interventions to generate and promote health literacy for its public health benefits (31). Recent reports indicate that governments and researchers are gradually turning attention toward not only addressing “…the practical challenges of low health literacy in clinical settings,” but also “describe approaches to improving health literacy in different clinical and community populations” [(31), p. 902]. Given recent global efforts toward promoting active and productive aging (33, 34), there is a need for health practitioners and researchers to have a better understanding of the health-related opportunities available to older persons, particularly on understudied issues such as health literacy, which provide people “…a route to greater autonomy and control over health decision-making” [(31), p. 902].

### Aims of the Study

**Figure 1:**
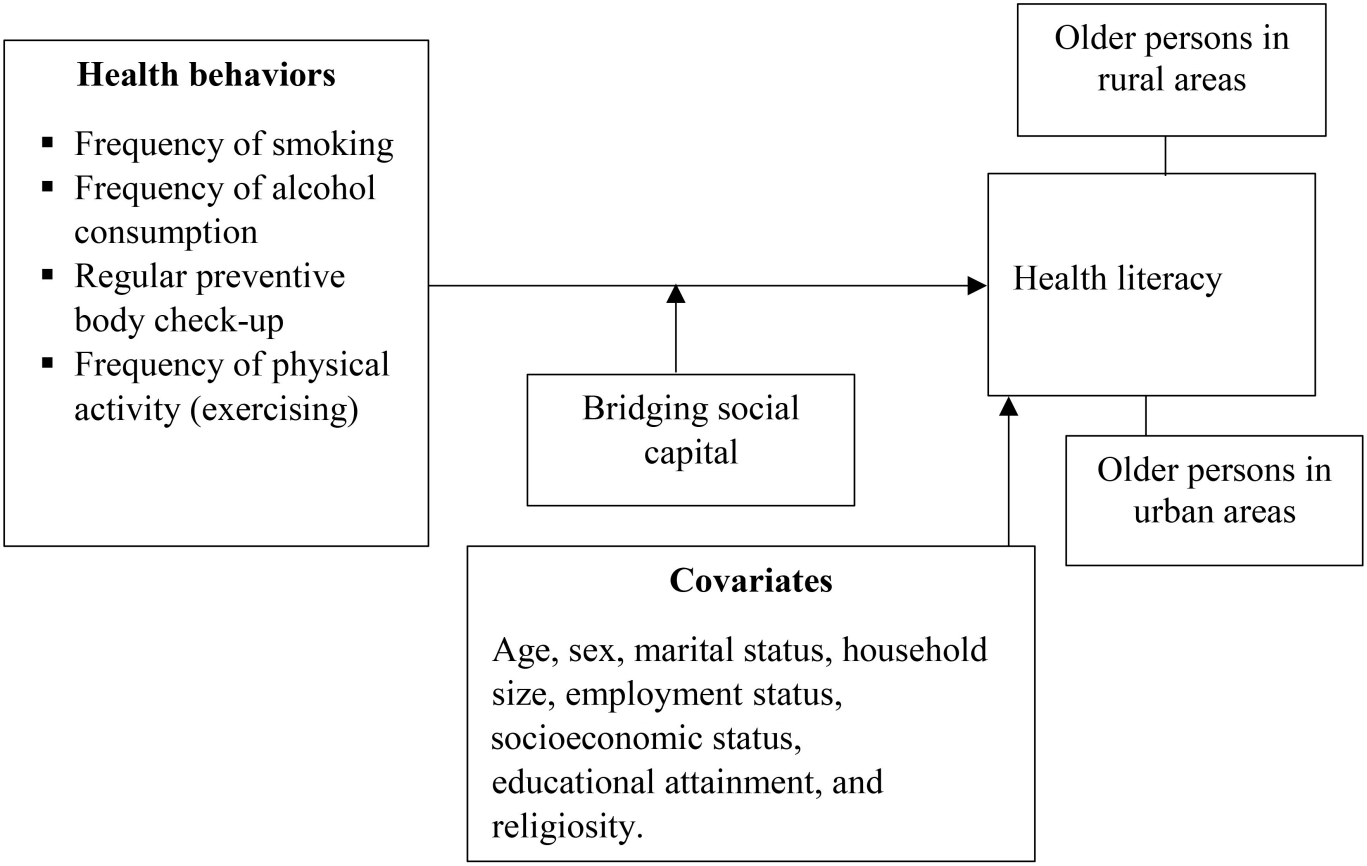
A heuristic framework of the study.

This study explores the moderating effect of weak social ties, referred to as ‘bridging social capital' on the associations between a set of health behaviors such as smoking, alcohol intake, body check-ups and physical exercise, and health literacy, as shown in Figure 1. The study of the influence of social ties contributes to the theoretical and empirical discourse about the social determinants of health literacy. Additionally, the study of the behavioral correlates of health literacy is a significant shift from the predominant approach of considering health literacy as a predictor of health and health-related behaviors (6–8). Thus, this study conceptualizes health literacy as an asset (or outcome) [see (2, 30)]. In addition to contributing to the existing empirical literature on health literacy research, this study offers another step toward establishing a causal association between health literacy and health-related outcomes by complementing existing studies that have examined the effect of health literacy on health behaviors in the literature. More importantly, by examining the specified relationships in Figure 1, this study will expand current health literacy research in Ghana and sub-Saharan Africa in general. This region has seen little focus on older persons, except for the work of Amoah (16).

### Health Behaviors, Social Capital, and Health Literacy Among Older Persons: Theoretical Perspectives and Literature Review

Having sufficient health literacy is associated with positive health behaviors among older adults (35–37). Older persons with sufficient health literacy are likely to be non-smokers, eat a balanced diet, abstain from alcohol, avoid sedentary lifestyles, and pay attention to their hygiene (8, 36, 37). However, some older persons lead a sedentary lifestyle and consume alcohol regularly despite having adequate health literacy (35). More importantly, the behavioral correlates of health literacy have geographical and several contextual dimensions (22, 23) that must be understood and incorporated in health literacy analyses of older persons. This points to a complex relationship between health behaviors and health literacy, which suggests a need to study the nature of older persons' health literacy from a multi-dimensional perspective. This is accomplished using the social-ecological model (26, 27, 38).

The social-ecological model places health outcomes, health-related behaviors, and health literacy in the context of the personal and socio-physical environments of people (26–28). It specifies five broad factors that shape health outcomes and health knowledge. These factors include individual capacities (e.g., knowledge, and educational attainment), interpersonal factors (i.e., social networks and support systems), institutional arrangements (e.g., organization of health services), community factors (social norms, practices, and beliefs of a place), as well as public policies (e.g., health-related policies) (26, 27, 39). Importantly, these factors interact and reinforce each other to affect health-related outcomes (26, 40). Thus, the social-ecological model offers a framework to empirically analyse the relationship between health behaviors and health literacy in a more holistic manner (26), as this study seeks to do. This study considers the socio-demographic characteristics (individual factors), health behaviors (individual and community factors), social capital (interpersonal factors), and geography (community factors) and their relations with health literacy (individual factors and, to some extent, community, and institutional factors).

Interpersonal characteristics such as social capital can affect how health behaviors are associated with health literacy (10, 22, 41). In this study, social capital refers to the resources (this can be information, tangible support, emotional support) that people receive or offer to others within their immediate and distant social networks, often because of the trusting relationships they possess (42–44). There are several types of social capital distinguished by the characteristics and strengths of the social networks involved. Specifically, resources emerging through weak ties are said to be more likely to promote changes in behavior compared to strong ties [see (42, 45)]. However, all of them have been found to influence health outcomes and behaviors significantly (43, 44, 46, 47). A unique aspect of social capital is bridging social capital, which concerns the resources emerging from weak social ties, including “a friend of a friend,” acquaintances, people of different socioeconomic status and class, and people of different religious and ethnic groups (42, 47). Bridging social capital can introduce new resources such as health information given its diversity in membership compared to, say bonding social capital (resources through family and close friends) (47). The diversity in bridging social capital makes it enormously influential in shaping health behaviors and health literacy. Having diverse sources of bridging social capital have been found in different places to negatively influence the relationship between smoking and health literacy (35, 48). However, extant evidence suggests that bridging social capital can propel people with low health literacy to behave in ways favorable to their health and access to accurate health information by tapping into the knowledge and resources of others (41).

Given these empirical observations and considering the spatial variations in the relations between social capital and health issues (24, 25), we hypothesize that bridging social capital will positively moderate the associations between health behaviors and health literacy among rural and urban residents. In Ghana, the components and effects of bridging social capital between rural and urban residents differ in terms of diversity in the socio-economic characteristics of people involved in the networks (25, 49). This implies that the interactions and process of reinforcement between health behaviors, health literacy, and bridging social capital as gauged through the social-ecological model are likely to differ relative to various health-related behaviors across rural and urban populations. Notwithstanding, the extent to which such differences affect health literacy and associated health behaviors have not received adequate attention in existing research, and this study fills this gap.

## Methods

### Study Design

The present study is part of a broader research project that examined the social aspects of health and wellbeing among youth, middle-aged and older persons in rural and urban Ghana. This study includes data from five of the former ten regions of Ghana. The five regions included Ashanti, Brong Ahafo, Greater Accra, Northern and Eastern Regions. Using a multi-stage cluster sampling approach, respondents were selected from 29 districts and 128 communities/areas (including 51 rural and 77 urban areas). The regions, districts and communities were purposively selected to obtain an adequate representation of rural and urban populations. This purposive approach ensured that regions, districts, and communities of different socioeconomic profiles (e.g., high and low economic areas), geographical (e.g., rural and urban areas and different parts of the country—southern, middle and northern parts; districts within the regions; and different areas of selected communities); and religious and ethnic profile of the country. A systematic sampling technique was used to select respondents to interview at the community level. This was to ensure a fair representation of the target populations in each community.

As part of the systematic sampling approach, an adult from every second house was interviewed in rural areas due to low population size and density compared to urban areas. Trained personnel interviewed one adult from every fifth house within various suburbs in the selected urban areas in line with related studies conducted in Ghana (50, 51). The choice of houses instead of households or individuals was meant to reduce similarities in responses as people who share the same housing arrangements tend to be similar in socioeconomic characteristics and experiences [see (50)]. The broader research on which the current study is based generated a sample of 2,097 using the formula *Ns* = (*Np*)(*p*)(1–*p*)/(*Np* −1)(*B*/*C*)^2^ + (*p*)(1–*p*); where Ns = total sample size needed; Np = size of the population; p = proportion expected to answer a certain way; B = acceptable level of sampling error; C = Z statistic associated with confidence interval. A confidence level of 95%, sample error of 0.05, and an assumption that 50% of respondents would provide consistent responses (52). Detailed information about the study design and sampling had been reported elsewhere (53, 54).

The study analyses the responses of 522 respondents, including 229 rural and 293 urban respondents. While the sample for each group is relatively low, the models constructed for the analyses were not overfitted as evaluated by the formula 50 + 8n (where n is the number of predictors) (55). With a maximum of 17 predictors (see **Table 2**) for all models, a minimum sample of 186 was required, but samples for both rural and urban areas exceeded the benchmark. This study focused on those who were 50 years and older. The age criterion is consistent with the practice of existing studies in Ghana and the relatively low life expectancy in Ghana and other developing countries [see (51)]. Written and, where necessary, oral informed consent was obtained from all respondents prior to enrolling them into the study. The Research Ethics Committee of Lingnan University approved the study protocol (EC-043/1718), while the Council for Scientific and Industrial Research (CSIR), Ghana provided in-country approval (RPN 005/CSIR-IRB/2018).

### Measures

#### Independent Variables

The focal independent variables, namely, health behaviors, was captured through four variables, including alcohol consumption, frequency of smoking, frequency of undertaking physical activities aside from work (i.e., exercising), and frequency of voluntary body check-up. Each of the variables was measured using one-item instruments. For the first three behaviors, respondents were asked how often they smoked, consumed four or more glasses of alcoholic drink, and undertook physical activities, aside from their routine activities or work, in the past 12 months. A five-point Likert scale which was coded as (1) “daily,” “several times a week,” “several times a month,” “once a month” and (5) “never” was used for all these questions. The responses were treated as ordinal variables in the regression analyses. Regarding voluntary body check-up, respondents were asked whether they had voluntarily visited a healthcare provider for a routine health check in the past 24 months preceding the study. Response to this question was (1) “yes” or (0) “no.”

#### Dependent Variable

Health literacy was measured using the 16-item European Health Literacy Questionnaire (HLS-EU-Q16). Variants of this instrument have been used in both developed and developing regions to measure health literacy among different population groups (56, 57). Respondents were asked the question: “how easy or difficult is it for you to”: (i) find information about treatments for illnesses that concern you; and (ii) understand what your doctor says to you. Their response was on a four-point Likert scale ranging from (1) “very difficult,” (2) “difficult,” (3) “easy,” to (4) “very easy.” To have a breakdown of the health literacy status of respondents, the responses were categorized as follows in the descriptive analyses: “very difficult” and “difficult” were scored as “0,” and “easy” and “very easy” scored “1.” A summative index was created, ranging from 0 to 16, with low values reflecting difficult and high values denoting easy. This conception of the dependent variable was then used in Ordinary Least Squares regression (OLS). The health literacy instrument had a Cronbach alpha of 0.85. For the purposes of conducting descriptive statistics, the data were classified as follows: inadequate health literacy (scores 0–8), problematic health literacy (scores 9–12), and sufficient health literacy (scores 13–16) (58).

#### Moderator

We used the Adapted Social Capital Assessment Tool (S-ASCAT) to measure bridging social capital (42). Respondents were given a list comprising several common examples of sources of bridging social capital and were asked to select as many as applicable (or state other sources) where they had received any form of support (information, tangible help, or emotional support) in the past 12 months. The responses were summed to form a bridging social capital score, with high scores representing more social capital and vice versa.

#### Covariates

We controlled for the following demographic and socioeconomic characteristics that the literature has shown to influence health literacy (23, 56, 57, 59): age (in completed years), sex (1-male and 0-female), household size (absolute number), subjective socioeconomic status (rated from 1, low to 10, high), educational attainment [1-never been to school (reference category), 2-primary school, 3-middle school, 4-secondary school including O'Level and A'Level, and 5-tertiary education], employment status (employed, pensioned/retired, unemployed), religiosity (seven-point scale from 1-extremely non-religious, very non-religious, somewhat non-religious, neither religious nor non-religious, somewhat religious, very religious, to 7-extremely religious), marital status (1-married and 0-unmarried), and region of residence [Ashanti (reference category), Brong Ahafo, Eastern, Northern, and Greater Accra regions]. We also measured their self-perceived health status (ranked from poor, fair, good, very good, to excellent). Table 1 contains more information about these variables.

**Table 1:** Descriptive statistics of the variables in the study by rural and urban respondents.

**Variable**	**Rural**		**Urban**		***p*** **-value**	**Overall**	
	**Mean/valid *n* (*N* = 229)**	**SD/%**	**Mean/valid *n* (*N* = 293)**	**SD/%**		**Mean/valid *n* (*N* = 522)**	**SD/%**
Age (in years)/Range	62.34/50–91	8.26	60.46/50–90	9.15	**0.015** ^a^	61.28/50–91	8.81
**Sex**					0.910		
Male	120	52.4	155	52.9		275	52.7
Female	109	47.6	138	47.1		247	47.3
**Household size**					0.942^a^		
Mean/Range	7.54/2–30	5.9	7.50/1–18	6.5		7.52/1–30	6.25
**Socioeconomic status (SES)**	4.1	1.8	4.3	1.8	0.246^a^	4.22	1.80
**Region of residence**					**0.000**		
Ashanti	73	31.9	62	21.2		135	26.9
Brong Ahafo	53	23.1	49	16.72		102	19.5
Northern region	62	27.11	60	20.48		122	23.4
Eastern region	41	17.9	57	19.45		98	18.8
Greater Accra	–	–	65	22.18		65	12.5
**Educational attainment**					**0.001**		
Never been to school	101	44.1	86	29.4		187	35.8
Primary school	56	24.5	63	21.5		119	22.8
Middle school	37	16.2	76	25.9		113	21.6
Secondary school	24	10.5	38	13.0		62	11.9
Tertiary	11	4.8	30	10.2		41	7.9
**Employment status**					**0.000**		
Employed	131	57.2	197	67.2		328	62.8
Pension/retired	24	10.5	50	17.1		74	14.2
Unemployed	74	32.3	46	15.7		120	23.0
**Marital status**					0.399		
Unmarried	67	29.3	76	25.9		143	27.4
Married	162	70.7	217	74.1		379	72.6
**Religiosity**					0.435^a^		
Mean (SD)	5.8 (1.0)		5.8 (1.1)			5.8 (1.1)	
Minimum–maximum	1–7		1–7			1–7	
**Health literacy (HL)**					**0.005** ^a^		
Inadequate	82	35.8	95	32.4		177	33.9
Problematic	113	49.3	125	42.7		238	45.6
Sufficient	34	14.8	73	24.9		107	20.5
Mean (SD)	9.1 (4.1)		10.1 (4.2)			9.7 (4.2)	
Minimum–Maximum score	0–16		0–16			0–16	
**Bridging social capital**					**0.000** ^a^		
Mean (SD)	1.6 (0.9)		2.0 (1.2)			1.8 (1.2)	
Minimum–maximum	0–5		0–5			0–5	
**Voluntary body check-up**					**0.002**		
Yes	51	22.2	101	34.5		152	29.1
No	178	77.8	192	65.52		370	70.9
**Alcohol intake**					0.312^a^		
Daily	12	5.2	9	3.1		21	4.0
Several times a week	8	3.5	8	2.7		16	3.1
Several times a month	8	3.5	17	5.8		25	4.8
Once a month or less often	42	18.3	46	15.7		88	16.9
Never	159	69.4	23	72.7		372	71.3
**Smoking**					0.982^a^		
Daily	4	1.7	12	4.1		16	3.1
Several times a week	16	7.0	9	3.1		25	4.8
Several times a month	11	4.8	22	7.5		33	6.3
Once a month or less often	14	6.1	3	1.0		17	3.3
Never	184	80.3	247	84.3		431	82.6
**Physical activities**					**0.000** ^a^		
Never	50	21.8	142	74.0		192	36.8
Once a month or less often	62	27.1	48	16.4		110	21.1
Several times a month	33	14.4	56	19.1		89	17.0
Several times a week	47	20.5	18	6.1		65	12.5
Daily	37	16.2	29	9.9		66	12.6
**Health status**					**0.000** ^a^		
Poor	37	16.2	33	11.3		70	13.4
Fair	101	44.1	107	36.5		208	39.8
Good	74	32.3	95	32.4		169	32.4
Very good	11	4.8	45	15.4		56	10.7
Excellent	6	2.6	13	4.4		19	3.6

*Bold figures: p < 0.05;*

a *p-value based on independent sample t-test. All other p-values are based on Chi-Square tests*.

### Data Analyses

The analyses comprised two parts. The first part involved descriptive statistics, which provided an overview of the respondents' sociodemographic characteristics, and their status as regards the independent, dependent, and moderating variables. A few variables had missing responses (mostly < 3% of total responses). These missing responses were replaced by the mean where necessary. Chi-square and independent samples *t*-test techniques were used to evaluate association and the differences between older persons in rural and urban areas with respect to the main variables used in the study. Spearman's rank correlation analyses for rural, urban, and overall populations were undertaken (see Appendix 1) to identify initial relations between all variables in the study and health literacy as the responses to the dependent variable were not normally distributed.

In the second part of the analysis, an OLS regression technique was used to evaluate the associations of health behaviors and bridging social capital with health literacy. This part also assessed the association between the interaction terms of each health behavior and bridging social capital with health literacy among rural and urban residents. The models for rural and urban residents comprised only variables that were significantly associated with health literacy in Spearman's rank correlation analyses. For each group of residents, two original regression models were constructed. The first included the covariates, and the second included the independent variables, moderators, and interaction terms. The interaction terms showing significant association with health literacy were further evaluated through slope analysis to identify the association between health behaviors and health literacy at different levels of bridging social capital. The evaluation was done at one standard deviation below and above the mean of bridging social capital to represent low and high levels, respectively. We used the software provided by Dawson (60) to prepare and test the simple slopes.

## Results

The respondents were aged 61 years on average, with the majority being males (54%), as shown in Table 1. There were more urban (56.1%) than rural residents (43.9%) in the sample. Urban residents were more likely to be educated and employed than their rural counterparts. The urban residents were more likely to have sufficient health literacy (24.9 vs. 14.8%) and higher bridging social capital (mean of 2.0 vs. 1.6) than rural residents. Urban residents were also more likely to undertake voluntary body check-ups (34.5 vs. 22.2%) and describe their health in more favorable terms than rural residents. However, older persons in rural areas were more likely to indulge in physical activities than urban residents (Table 1).

In Table 2, model 1 represents the main effects and model 2 includes the interaction terms. According to model 2, none of the health behavior variables assessed was directly associated with the health literacy of both urban and rural residents. However, the main effects in Model 1 show that smoking (*B* = −0.787, *p* < 0.05) was negatively associated with health literacy and undertaking physical activities (*B* = 0.812, *p* < 0.01) had a positive association with health literacy among rural older persons. This indicates that the more an older person smoked, the lower their health literacy. In contrast, regularly indulging in physical activities was an indication of higher health literacy.

**Table 2:** Socio-demographic and behavioral factors associated with health literacy of older persons in rural and urban areas^a^.

	**Rural**				**Urban**				**Overall**			
	**Model 1**		**Model 2**		**Model 1**		**Model 2**		**Model 1**		**Model 2**	
	***B (95% CI)***	**Std. error**	***B (95% CI)***	**Std. error**	***B (95% CI)***	**Std. error**	***B (95% CI)***	**Std. error**	***B (95% CI)***	**Std. error**	***B (95% CI)***	**Std. error**
Age	−0.030 (−0.108, 0.048)	0.039	−0.037 (−0.114, 0.040)	0.039	–	–	–	–	−0.001 (−0.011, 0.010)	0.005	−0.001 (−0.011, 0.010)	0.005
**Sex**												
Males	0.634 (−0.598, 1.867)	0.625	0.877 (−0.356, 2.109)	0.625	0.219 (−0.020, 0.459)	0.122	0.205 (−0.032, 0.441)	0.120	0.136 (−0.035, 0.306)	0.087	0.136 (−0.035, 0.307)	0.087
Female (ref)												
**Employment status**												
Pension (Retired)	1.185 (−0.873, 3.242)	1.044	0.882 (−1.177, 2.941)	1.044	−0.328 (−0.713, 0.057)	0.195	−0.316 (−0.701, 0.069)	0.195	0.125 (−0.157, 0.407)	0.145	0.122 (−0.161, 0.404)	0.144
Unemployed	−1.101 (−3.322, 1.120)	1.127	−1.706 (−3.957, 0.544)	1.141	−1.241 (−1.692, −0.791)^***^	0.228	−1.296 (−1.747, −0.845)^***^	0.229	−0.549 (−0.853, −0.245)^***^	0.156	−0.554 (−0.859, −0.249)^***^	0.155
Employed (ref)												
**Marital status**												
Married	–	–	–	–	−0.005 (−0.281, 0.272)	0.140	−0.029 (−0.302, 0.244)	0.139	0.002 (−0.183, 0.187)	0.087	0.004 (−0.182, 0.189)	0.094
Unmarried (ref)												
**Educational attainment**												
Primary school only	1.240 (−0.322, 2.802)	0.792	1.633 (0.077, 3.190)^*^	0.789	–	–	–	–	0.041 (−0.170, 0.252)	0.107	0.041 (−0.170, 0.252)	0.107
Middle school	3.357 (−0.789, 7.503)	2.103	3.643 (−0.438, 7.723)	2.069	–	–	–		−0.023 (−0.318, 0.271)	0.150	−0.030 (−0.326, 0.266)	0.150
Secondary school	4.110 (2.397, 5.823)^**^	0.869	4.615 (2.873, 6.357)^***^	0.884	–	–	–		0.365 (0.103, 0.627)^**^	0.133	0.366 (0.103, 0.628)	0.133
Tertiary educational attainment	3.291 (0.654, 5.927)^*^	1.337	3.041 (0.440, 5.643)^*^	1.320	–	–	–		0.714 (0.369, 1.060)	0.176	0.711 (0.365, 1.057)	0.176
Never been to school (ref)												
SES	–	–	–	–	0.013 (−0.046, 0.071)	0.030	0.012 (−0.046, 0.070)	0.029	0.001 (−0.044, 0.045)	0.023	0.001 (−0.044, 0.046)	0.023
Religiosity	1.070 (0.488, 1.653)^***^	0.295	1.015 (437, 1.593)^***^	0.293	0.040 (−0.061, 0.140)	0.051	0.032 (−0.067, 0.132)	0.051	0.062 (−0.011, 0.135)	0.037	0.0625 (−0.008, 0.135)	0.037
Overall health status	0.826(0.207, 1.445)^**^	0.314	0.705(0.090, 1.320)^*^	0.318	0.256(0.137, 0.375)^***^	0.060	0.261(0.143, 0.378)^***^	0.060	0.175(0.083, 0.266)^***^	0.046	0.168(0.077, 0.260)^***^	0.046
Bridging SC	0.315(−0.974, 0.345)	0.334	1.354(−3.877, 6.584)	2.652	−0.049(−0.157, 0.059)	0.055	−0.997(−1.685, −0.310)^**^	0.349	−0.014(−0.098, 0.071)	0.043	−0.046(−0.200, 0.108)	0.078
Smoking	−0.787(−1.396, −0.178)^*^	0.309	−0.973(−1.376, 1.358)	0.693	–	–	–	–	–	–	–	–
Physical activities	0.812(0.207, 1.416)^**^	0.307	0.009(−1.285, 1.548)	0.718	–	–	–	–	0.012(−0.068, 0.092)	0.041	−0.0297(−0.184, 0.126)	0.079
Alcohol intake	0.170(−0.419, 0.759)	0.299	−0.288(−1.809, 1.233)	0.771	−0.097(−0.220, 0.026)	0.062	−0.309(−0.646, 0.028)	0.171	–	–	–	–
Voluntary body check–up	–	–	–	–	0.155(0.044, 0.265)^**^	0.056	−0.008(−0.228, 0.211)	0.111	–	–	–	–
Alcohol^*^Bridging social capital			1.770(−2.884, 6.424)	2.360			0.185(0.043, 0.328)^*^	0.072	–	–	–	–
Smoking^*^Bridging social capital			−5.032(−8.417, −1.648)^**^	1.717	–	–	–	–	–	–	–	–
Physical activity^*^Bridging social capital			1.601(−0.837, 4.040)	1.237	–	–	–	–			0.073(−0.163, 0.309)	0.120
Routine body check-up^*^Bridging social capital	–	–	–	–			0.192(−0.036, 0.419)	0.116	–	–	–	–
Adjusted *R*-Square	0.333		0.357		0.284		0.304		0.320		0.319	

* *p < 0.05,*

** *p < 0.01,*

*** *p < 0.001.*

a *Controlled for region of residence;*

− *Variables not included in the model due to lack of correlation with health literacy*.

At the same time, having voluntary body check-ups (*B* = 0.155, *p* < 0.01) was positively associated with health literacy of urban older persons. Thus, older persons who voluntarily undertook body-check-ups were likely to have high scores in health literacy. It was also found that having more sources of bridging social capital tended to indicate a lower level of health literacy among urban residents (*B* = −0.997, *p* < 0.01). There was no association between bridging social capital and health literacy among rural residents (Table 2, Model 2).

**Figure 2:**
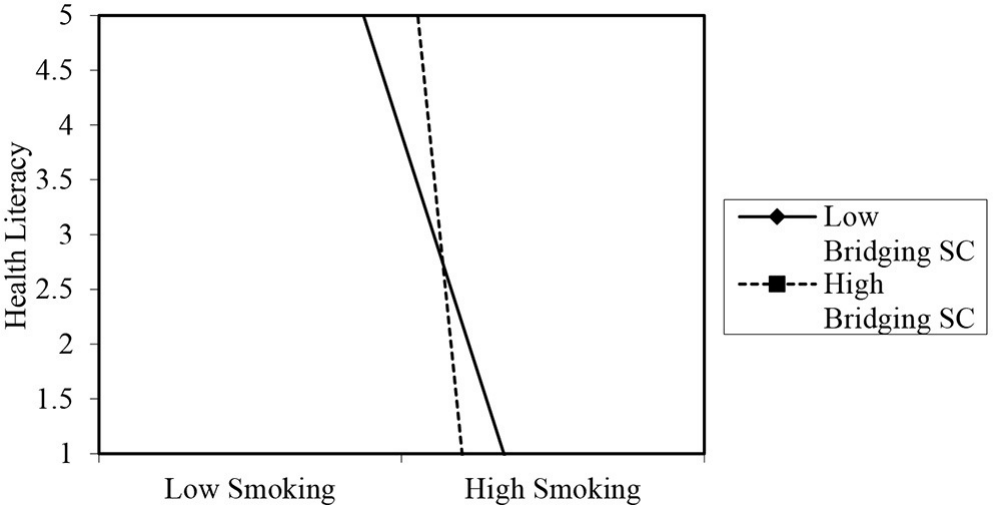
Moderating role of bridging social capital in the association between smoking and health literacy of rural older persons. The graph merely projects the relationship. The values on the y-axis are for projection of the figure only. Source of template and accompanying statistics: Dawson (60).

**Figure 3:**
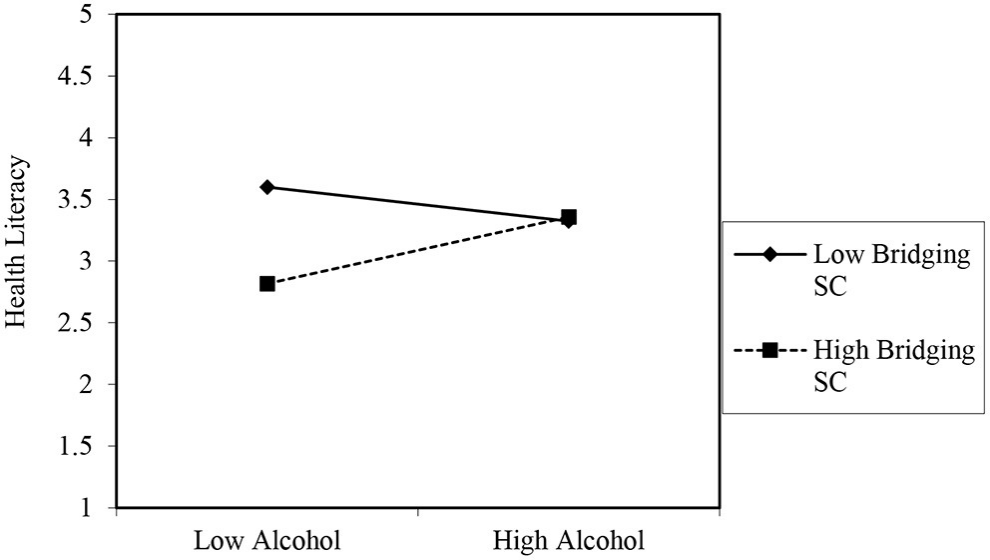
Moderating role of bridging social capital in the association between frequent alcohol intake and health literacy of urban older persons. The graph merely projects the relationship. The values on the y-axis are for projection of the figure only. Source of template and accompanying statistics: Dawson (60).

Bridging social capital negatively moderated the association of smoking with health literacy among older persons in rural areas (*B* = −5.032, *p* < 0.01). This was confirmed in a subsequent simple slope test (see Figure 2), which showed that when an older person has access to more bridging social capital, it reduces the rate at which the person smokes, which indicates an increased level of health literacy (*B* = −15.817, *t* = −3.052, *p* = 0.000) as evaluated by the resource provided by Dawson (60). In contrast, a non-significant relationship was observed between smoking and health literacy at low levels of bridging social capital (*B* = −4.828, *t* = −1.762, *p* = 0.080). Among older persons in urban areas, having greater access to sources of bridging social capital was significantly associated with more alcohol intake, which points to low health literacy (*B* = 0.185, *p* < 0.05). Indeed, the simple slope analysis showed that when bridging social capital is high, frequent (i.e., high) alcohol intake was an indication of high health literacy (*B* = 0.285, *t* = 4.828, *p* = 0.000). However, when bridging social capital was low, a negative relationship (albeit non-significant) between alcohol intake and health literacy was observed. Thus, older persons with low bridging social capital were likely to take less alcohol which indicated a propensity for higher health literacy (*B* = −0.145, *t* = −1.345, *p* = 0.180) as depicted in Figure 3.

## Discussion

This study explored the socio-ecological dimensions of the associations between health-related behaviors and health literacy. We found evidence that health-related behaviors (e.g., smoking, undertaking physical activities, and voluntary body-check-up) and bridging social capital are associated with health literacy among older persons in both rural and urban areas. Our hypothesis on the moderating role of bridging social capital returned mixed results as negative and positive influences were observed. Theoretically, the results of this study support the central premise of the social-ecological theory that a multi-dimensional approach to analyzing health literacy and health-related issues are likely to improve our understanding of the personal, sociocultural and institutional context of the phenomenon and offer opportunities for intervention using contextually relevant and evidenced-based approaches (26). The ensuing discussions expand on this standpoint, given the specific findings from the study.

The preliminary results showed that urban older persons were more likely to have sufficient health literacy than those in rural areas. Residents in urban areas in Ghana tend to be more educated; are exposed to proper health information; and have relatively better healthcare services than those in rural areas (22). These favorable conditions ensure that older persons in urban areas are more likely to access accurate health information about preventive and curative health issues. The higher health literacy level may explain health-promoting behaviors such as voluntary body check-ups and older persons' overall positive self-reported health in urban areas. Indeed, this study found evidence that behaviors such as voluntary body check-ups are positively associated with health literacy among older persons in urban areas. This positive association correlates with the observation of those in rural areas where smoking and engaging in physical activities were negatively and positively associated with health literacy, respectively. These positive associations are consistent with existing literature (2, 6, 36). While most existing research has presented behaviors as a consequence of health literacy (2, 30, 61), our analyses point to an alternative way of understanding the relations between health literacy and health behaviors by considering health literacy as an asset. This empirical observation lends support to extant theoretical proposals about a causal relationship between health literacy and health-related wellbeing (32). Health behaviors can be an important indicator of health literacy status among older persons in urban areas. Future research should explore evidenced-based interventions to promote positive health behaviors as part of overarching measures to improve health literacy among older persons.

Regarding social capital, older persons in urban areas had comparatively more sources of bridging social capital than their rural counterparts, contrary to what Ziersch et al. (24) observed that rural residents in South Australia have high higher levels of close and distant social networks than urban residents. A potential reason for the difference is the heterogeneity (e.g., in terms of ethnicity, religious affiliations, professional backgrounds of residents) of urban societies vis-à-vis rural areas in Ghana (62). Unlike urban areas, rural societies in most places, including high-income countries, tend to be close-knit. This implies that rural residents generally have homogenous social contacts and less diverse memberships. In Ghana, many rural communities comprise several extended families (22). Such familial composition and other characteristics of rural settings such as low access to health and educational services and professionals exemplify why bridging social capital was low in this study (25, 62).

This study found that bridging social capital negatively moderated the association of smoking with health literacy among older persons in rural areas. Existing studies indicate that bridging social capital limits poor health choices such as smoking while helping others quit smoking successfully through supportive environments (63, 64). Bridging social capital has also been shown to be instrumental in lowering the incidence of health-compromising behaviors (63). While other existing evidence suggests that more social contact can spur behaviors such as smoking due to social pressures (35), this study shows that bridging social capital can promote positive health behaviors among older persons in rural settings. In the context of Ghana, it is documented that some sociocultural norms and practices strongly abhor corrosive lifestyle choices such as smoking and alcohol intake (22, 65, 66). Such norms are dominant in rural settings, and their influence may explain why bridging social capital is sometimes considered as a community attribute (22, 67). Because of the importance of social norms and practices to social relationships and their functions, it is vital that interpretation of the association between health behavior and health literacy be made cautiously as instances of no association were also observed in the study. It is possible that factors relating to social norms and normative practices are stronger determinants of health literacy than health behaviors. Notwithstanding such instances of no association between health behaviors and health literacy of older persons, the study's findings are consistent with research elsewhere (8, 37).

Unlike rural areas, bridging social capital strengthened the association between alcohol intake and health literacy among older persons in urban areas. This is contrary to the hypothesis and the findings of other studies (63, 64). This contrary finding requires in-depth study, particularly using qualitative methods to explore how bridging social capital could potentially increase the propensity of older persons to abuse alcohol despite sufficient health literacy. However, this finding is not unique as other studies surmise that more social contacts can spur behaviors such as smoking due to social pressures (35). Access to diverse social support through different networks that bridging social capital embodies also means that the propensity of negative influence increases (68). This is likely the case among older persons in urban areas in this study, where bridging social capital was higher than rural areas. Obviously, some of those negative influences are empirically exhibited among the older persons in urban areas in this study. This study's findings imply that the value of bridging social capital to how health behaviors relate to older persons' health literacy in urban areas must be assessed in terms of quality (i.e., the value of the support/influence that is generated) instead of focusing on the quantity of support alone (69). Moreover, the inconsistency in the role of bridging social capital in the association of health behaviors and health literacy of older persons, as demonstrated in this study, invites researchers to engage more deeply with the research question across contexts in order to isolate the specific instances in which negative and positive impacts are observed.

These results and arguments emphasize the tenets of the social-ecological theory of health by echoing that health promotion strategies concerning health literacy, and even health behaviors, should not focus on individuals alone. Issues of health-related knowledge and behaviors should be analyzed from a social-ecological perspective. Such perspective allows for consideration of factors at micro, meso and macro levels of society in analyses. Analytical approaches that include these multi-contextual level factors can help inform practices and policies for improving health literacy as part of active aging programmes and measures to achieve global targets such as the third Sustainable Development Goal that deals with good health and wellbeing (70). Efforts to improve health literacy are, however, not an end in themselves but rather a significant step toward reducing health inequities at different levels of societies through good governance, strong local action (at cities and communities levels), and people's empowerment (70). In view of this, the findings from this study provide an important reminder of how local and inter-personal factors such as weak social ties can shape how health literacy can be improved. For instance, while the World Health Organization advocates for inclusive and equitable access to quality education and life-long learning to improve health literacy (70), this study's findings indicate that such strategies are unlikely to be successful if local factors such as bridging social capital are not properly addressed and incorporated into such approaches.

### Limitations

This is the only study examining the association between health behaviors and health literacy among older persons in rural and urban Ghana to the best of our knowledge. While our results are instructive for interventions promoting health efficacy and active aging of older persons, they must be interpreted cautiously. The analyses in this study are based on a cross-sectional survey which makes it difficult to attribute causation. Also, given that the responses are based on subjective evaluations and reports of respondents, it is likely that some respondents may have responded in a manner that corresponds with the prevailing social expectations. For example, some respondents may have under-reported the frequency of their smoking or alcohol intake, which can affect the study's conclusions.

## Conclusions

This study examined the association between health behaviors and health literacy and explored whether bridging social capital can modify these relations among rural and urban older persons in Ghana. We found that older persons in urban areas have higher health literacy and bridging social capital than their rural counterparts.

The study has provided empirical evidence of the association between health behaviors and health literacy among older persons in rural and urban Ghana. While research and practice have often considered health behavior an outcome of health literacy, this study shows that a reverse of such analyses achieves consistent results. Perhaps more importantly, the findings can help identify older persons at risk of low health literacy in rural and urban areas by focusing on their common health-related behaviors. Such knowledge can help improve the health literacy of older persons as part of interventions that consider health literacy as a public health asset. However, such potential interventions must take cognisance of the inconsistent observation that has been made regarding the moderating role of bridging social capital in the association between health behaviors and health literacy. Bridging social capital negatively moderated the association between smoking and health literacy in rural areas but positively modified the relationship between alcohol intake and health literacy in urban areas. Thus, for policymakers and practitioners aiming to promote older persons' health literacy as a public health asset at individual and community levels, an important starting point to achieving such goals is to understand the fundamental indicators (e.g., health behaviors) and the role that social and geographical factors play in shaping their health literacy. For instance, if well-generated and tailored, support through weak social ties (i.e., bridging social capital) can be an important resource for positive behavioral change as part of health literacy interventions among older persons in rural and urban settings.

## Data Availability Statement

The data supporting the conclusions of this article will be made available by the authors upon request on case by case basis.

## Ethics Statement

The Research Ethics Committee of Lingnan University approved the study protocol (EC-043/1718), while the Council for Scientific and Industrial Research (CSIR), Ghana provided in-country approval (RPN 005/CSIR-IRB/2018). The respondents provided their written or oral informed consent to participate in this study.

## Author Contributions

PA conceived this study and took the lead in preparing the manuscript including data analyses. JM provided support in data analyses and presentation and discussion. KA supported the study through literature review and drafting the background of the article. All authors contributed to the article and approved the submitted version.

## Funding

This work was supported by the Lingnan University Faculty Grant (102159) and Lam Woo Research Fund-Individual Grant (Lingnan University) (LWI20014).

## Conflict of Interest

The authors declare that the research was conducted in the absence of any commercial or financial relationships that could be construed as a potential conflict of interest.

## Publisher's Note

All claims expressed in this article are solely those of the authors and do not necessarily represent those of their affiliated organizations, or those of the publisher, the editors and the reviewers. Any product that may be evaluated in this article, or claim that may be made by its manufacturer, is not guaranteed or endorsed by the publisher.
