# Supplementary material for: Health Behaviors and Health Literacy: Questing the Role of Weak Social Ties Among Older Persons in Rural and Urban Ghana
Source: Front Public Health. 2022 Feb 28;10:777217. doi: 10.3389/fpubh.2022.777217 (PMC8919952; doi:10.3389/fpubh.2022.777217)
Supplement: Supplementary file 1 [file Data_Sheet_1.docx]

**Health behaviours and health literacy: Questing the role of weak social ties among older persons in rural and urban Ghana**

# Appendix 1:

## Table 1: Spearman’s correlation analysis of variables in the overall sample of the study - Overall Sample

|  |  | 1 | 2 | 3 | 4 | 5 | 6 | 7 | 8 | 9 | 10 | 11 | 12 | 13 | 14 | 15 | 16 | 17 |
| --- | --- | --- | --- | --- | --- | --- | --- | --- | --- | --- | --- | --- | --- | --- | --- | --- | --- | --- |
|  | Health Literacy | 1.000 |  |  |  |  |  |  |  |  |  |  |  |  |  |  |  |  |
|  | Region | .339^**^ | 1.000 |  |  |  |  |  |  |  |  |  |  |  |  |  |  |  |
|  | Rural/urban | -0.032 | -.181^**^ | 1.000 |  |  |  |  |  |  |  |  |  |  |  |  |  |  |
|  | Age | -.087^*^ | .102^*^ | -.148^**^ | 1.000 |  |  |  |  |  |  |  |  |  |  |  |  |  |
|  | Sex | -.250^**^ | -.188^**^ | -0.005 | -0.058 | 1.000 |  |  |  |  |  |  |  |  |  |  |  |  |
|  | Household size | 0.019 | .377^**^ | -0.029 | 0.040 | -.186^**^ | 1.000 |  |  |  |  |  |  |  |  |  |  |  |
|  | Marital status | .149^**^ | .114^**^ | 0.049 | -.256^**^ | -.111^*^ | .125^**^ | 1.000 |  |  |  |  |  |  |  |  |  |  |
|  | Educational attainment | .159^**^ | -.301^**^ | 0.048 | -.146^**^ | -.120^**^ | -.244^**^ | -0.045 | 1.000 |  |  |  |  |  |  |  |  |  |
|  | Employment status | -.310^**^ | -0.073 | -.122^**^ | .319^**^ | 0.056 | .091^*^ | -.107^*^ | -.206^**^ | 1.000 |  |  |  |  |  |  |  |  |
|  | SES | -.093^*^ | -.479^**^ | 0.074 | -0.077 | 0.008 | -.173^**^ | -0.051 | .413^**^ | 0.026 | 1.000 |  |  |  |  |  |  |  |
|  | Religiosity | .126^**^ | .149^**^ | 0.074 | -0.064 | 0.003 | -0.017 | -0.003 | -0.042 | -.182^**^ | -0.033 | 1.000 |  |  |  |  |  |  |
|  | Physical activities | .117^**^ | .108^*^ | -.259^**^ | -.106^*^ | -.178^**^ | 0.024 | 0.036 | .156^**^ | -0.036 | 0.002 | -0.025 | 1.000 |  |  |  |  |  |
|  | Routine body check-up | 0.076 | -.129^**^ | .104^*^ | 0.011 | 0.005 | -0.078 | 0.021 | 0.042 | -0.052 | .092^*^ | .095^*^ | -.121^**^ | 1.000 |  |  |  |  |
|  | Alcohol | -0.052 | 0.040 | 0.037 | 0.084 | .205^**^ | 0.030 | -0.016 | -.173^**^ | .102^*^ | 0.044 | .170^**^ | -.217^**^ | 0.052 | 1.000 |  |  |  |
|  | Smoking SC | -0.073 | -.234^**^ | 0.043 | -0.002 | .215^**^ | -.182^**^ | -0.022 | 0.081 | -0.053 | .103^*^ | 0.004 | -0.079 | -0.002 | .251^**^ | 1.000 |  |  |
|  | Bridging SC | .100^*^ | -0.041 | .181^**^ | -0.015 | -.101^*^ | 0.019 | 0.010 | 0.014 | -.127^**^ | -0.038 | .105^*^ | -0.060 | 0.086 | -0.037 | -0.065 | 1.000 |  |
|  | Health status | .285^**^ | 0.052 | .155^**^ | -.276^**^ | -.143^**^ | 0.084 | .134^**^ | 0.084 | -.097^*^ | 0.043 | 0.030 | -0.002 | 0.082 | -0.038 | -0.066 | -0.047 | 1.000 |

**Correlation is significant at the 0.01 level (2-tailed). *Correlation is significant at the 0.05 level (2-tailed)

## Table 2: Spearman’s correlation analysis of variables in the study - Rural

|  |  | 1 | 2 | 3 | 4 | 5 | 6 | 7 | 8 | 9 | 10 | 11 | 12 | 13 | 14 | 15 | 16 |
| --- | --- | --- | --- | --- | --- | --- | --- | --- | --- | --- | --- | --- | --- | --- | --- | --- | --- |
|  | Health Literacy | 1.000 |  |  |  |  |  |  |  |  |  |  |  |  |  |  |  |
|  | Region | .200** | 1.00 |  |  |  |  |  |  |  |  |  |  |  |  |  |  |
|  | Age | -.218** | 0.123 | 1.000 |  |  |  |  |  |  |  |  |  |  |  |  |  |
|  | Sex | -.307** | -.316** | -0.015 | 1.000 |  |  |  |  |  |  |  |  |  |  |  |  |
|  | Household Size | 0.053 | .522** | 0.028 | -.201** | 1.000 |  |  |  |  |  |  |  |  |  |  |  |
|  | Marital status | 0.118 | 0.004 | -.219** | -0.026 | 0.109 | 1.000 |  |  |  |  |  |  |  |  |  |  |
|  | Education | .275** | -.341** | -.326** | -0.124 | -.254** | 0.077 | 1.000 |  |  |  |  |  |  |  |  |  |
|  | Employment status | -.318** | -0.104 | .261** | 0.108 | 0.113 | -0.026 | -.292** | 1.000 |  |  |  |  |  |  |  |  |
|  | SES | -0.049 | -.534** | -0.116 | 0.061 | -.155* | 0.053 | .408** | 0.100 | 1.000 |  |  |  |  |  |  |  |
|  | Religiosity | .145* | .289** | -0.050 | -0.088 | 0.046 | -0.022 | -0.093 | -.197** | -0.118 | 1.000 |  |  |  |  |  |  |
|  | Physical activities | .209** | .326** | -.219** | -.220** | 0.016 | 0.071 | .135* | -0.056 | -0.085 | -0.034 | 1.000 |  |  |  |  |  |
|  | Check-up | 0.003 | -0.033 | 0.056 | 0.035 | -0.019 | 0.006 | -0.031 | -0.022 | 0.047 | .272** | -.187** | 1.000 |  |  |  |  |
|  | Alcohol | -.255** | -.163* | 0.120 | .365** | -0.110 | -0.105 | -.318** | .224** | 0.012 | 0.120 | -.309** | 0.050 | 1.000 |  |  |  |
|  | Smoking | -.260** | -.420** | -0.109 | .282** | -.368** | 0.018 | .147* | -.135* | 0.108 | -0.068 | -0.077 | -0.090 | .170** | 1.00 |  |  |
|  | Bridging SC | 0.199* | .314** | -0.002 | -0.116 | .151* | -0.022 | -0.002 | -.197** | -.167* | .351** | 0.104 | 0.114 | 0.083 | -0.051 | 1.000 |  |
|  | Health status | .287** | -0.007 | -.190** | -0.032 | .143* | 0.025 | .193** | 0.067 | 0.053 | -0.048 | 0.093 | 0.028 | -.163* | -0.095 | -.175** | 1. |

**Correlation is significant at the 0.01 level (2-tailed). *Correlation is significant at the 0.05 level (2-tailed)

## Table 3: Spearman’s correlation analysis of variables in the study - Urban

|  |  | 1 | 2 | 3 | 4 | 5 | 6 | 7 | 8 | 9 | 10 | 11 | 12 | 13 | 14 | 15 | 16 |
| --- | --- | --- | --- | --- | --- | --- | --- | --- | --- | --- | --- | --- | --- | --- | --- | --- | --- |
|  | CHL | 1.000 |  |  |  |  |  |  |  |  |  |  |  |  |  |  |  |
|  | Region | .381^**^ | 1.000 |  |  |  |  |  |  |  |  |  |  |  |  |  |  |
|  | Age | -0.007 | 0.022 | 1.000 |  |  |  |  |  |  |  |  |  |  |  |  |  |
|  | Sex | -.209^**^ | -.118^*^ | -0.103 | 1.000 |  |  |  |  |  |  |  |  |  |  |  |  |
|  | Household size | -0.036 | .333^**^ | 0.094 | -.142^*^ | 1.000 |  |  |  |  |  |  |  |  |  |  |  |
|  | Marital status | .171^**^ | .210^**^ | -.265^**^ | -.182^**^ | .135^*^ | 1.000 |  |  |  |  |  |  |  |  |  |  |
|  | Education | 0.089 | -.314^**^ | -0.009 | -0.109 | -.273^**^ | -.156^**^ | 1.000 |  |  |  |  |  |  |  |  |  |
|  | Employment status | -.330^**^ | -.133^*^ | .330^**^ | 0.018 | 0.038 | -.181^**^ | -.120^*^ | 1.000 |  |  |  |  |  |  |  |  |
|  | SES | .123^*^ | .486^**^ | -0.036 | -0.034 | -.239^**^ | -.146^*^ | .432^**^ | -0.015 | 1.000 |  |  |  |  |  |  |  |
|  | Religiosity | .121^*^ | 0.116 | -0.048 | 0.086 | -0.032 | 0.008 | 0.000 | -.139^*^ | 0.036 | 1.000 |  |  |  |  |  |  |
|  | Physical activities | 0.069 | -0.067 | -0.099 | -.153^**^ | -0.051 | 0.019 | .225^**^ | -.116^*^ | .120^*^ | 0.018 | 1.000 |  |  |  |  |  |
|  | Routine body check-up | 0.106* | -.149^*^ | 0.004 | -0.017 | -0.079 | 0.022 | 0.087 | -0.053 | 0.114 | -0.065 | 0.001 | 1.000 |  |  |  |  |
|  | Alcohol | -0.193* | .150^**^ | 0.055 | 0.075 | .145^*^ | 0.061 | -0.055 | 0.003 | 0.057 | .208^**^ | -.186^**^ | 0.047 | 1.000 |  |  |  |
|  | Smoking | -0.061 | -.129^*^ | 0.079 | .160^**^ | -0.025 | -0.060 | 0.029 | 0.042 | 0.101 | 0.062 | -0.054 | 0.058 | .316^**^ | 1.000 |  |  |
|  | Bridging SC | 0.100 | -.186^**^ | 0.033 | -0.093 | -0.022 | 0.012 | 0.022 | -0.042 | 0.036 | -0.104 | -0.095 | 0.034 | -.120^*^ | -0.089 | 1.000 |  |
|  | Health status | .281^**^ | 0.102 | -.290^**^ | -.223^**^ | 0.054 | .209^**^ | -0.021 | -.208^**^ | -0.006 | 0.094 | 0.003 | 0.089 | -0.033 | -0.044 | 0.004 | 1.000 |

**Correlation is significant at the 0.01 level (2-tailed). *Correlation is significant at the 0.05 level (2-tailed)
